# Episomal and chromosomal DNA replication and recombination in *Entamoeba histolytica*

**DOI:** 10.3389/fmolb.2023.1212082

**Published:** 2023-06-08

**Authors:** Sudha Bhattacharya

**Affiliations:** Department of Biology, Ashoka University, Sonipat, Haryana, India

**Keywords:** entamoeba histolytica, replication, homologous recombination, meiotic genes, ribosomal DNA episome, Entamoeba invadens, encystation

## Abstract

*Entamoeba histolytica* is the causative agent of amoebiasis. DNA replication studies in *E. histolytica* first started with the ribosomal RNA genes located on episomal circles. Unlike most plasmids, *Entamoeba histolytica* rDNA circles lacked a fixed origin. Replication initiated from multiple sites on the episome, and these were preferentially used under different growth conditions. In synchronized cells the early origins mapped within the rDNA transcription unit, while at later times an origin in the promoter-proximal upstream intergenic spacer was activated. This is reminiscent of eukaryotic chromosomal replication where multiple potential origins are used. Biochemical studies on replication and recombination proteins in *Entamoeba histolytica* picked up momentum once the genome sequence was available. Sequence search revealed homologs of DNA replication and recombination proteins, including meiotic genes. The replicative DNA polymerases identified included the α, δ, *ε* of polymerase family B; lesion repair polymerases Rev1 and Rev3; a translesion repair polymerase of family A, and five families of polymerases related to family B2. Biochemical analysis of EhDNApolA confirmed its polymerase activity with expected kinetic constants. It could perform strand displacement, and translesion synthesis. The purified EhDNApolB2 had polymerase and exonuclease activities, and could efficiently bypass some types of DNA lesions. The single DNA ligase (EhDNAligI) was similar to eukaryotic DNA ligase I. It was a high-fidelity DNA ligase, likely involved in both replication and repair. Its interaction with EhPCNA was also demonstrated. The recombination-related proteins biochemically characterized were EhRad51 and EhDmc1. Both shared the canonical properties of a recombinase and could catalyse strand exchange over long DNA stretches. Presence of Dmc1 indicates the likelihood of meiosis in this parasite. Direct evidence of recombination in *Entamoeba histolytica* was provided by use of inverted repeat sequences located on plasmids or chromosomes. In response to a variety of stress conditions, and during encystation in *Entamoeba invadens,* recombination-related genes were upregulated and homologous recombination was enhanced. These data suggest that homologous recombination could have critical roles in trophozoite growth and stage conversion. Availability of biochemically characterized replication and recombination proteins is an important resource for exploration of novel anti-amoebic drug targets.

## 1 Introduction

*Entamoeba histolytica* is a parasitic protist that causes amoebic dysentery and liver abscess, which if not timely treated can be fatal (Stanley, 2003). The parasite spreads by the faecal-oral route and is ingested through food and water contaminated with *E. histolytica* cysts. These migrate to the colon and get converted to trophozoites that rapidly multiply in the lumen. In asymptomatic infections trophozoites get re-converted to cysts which are excreted to complete the life cycle. In a minority of individuals, the trophozoites become invasive and invade the colonic epithelium causing intestinal ulcers, and may invade other organs, mainly the liver to form liver abscesses.

*Entamoeba histolytica* is an early-branching eukaryote with interesting unique features. It lacks typical mitochondria that are replaced by mitosomes which lack DNA, and may perform functions like sulfate activation and oxygen detoxification (van der Giezen and Tovar, 2005; Mi-ichi et al., 2009; Maralikova et al., 2010). Its ∼20 Mb genome is exclusively nuclear-localized (Lorenzi et al., 2010) and is composed of a number of repetitive elements. The non-long terminal repeat (LTR) retrotransposons, *E. histolytica* long and short interspersed nuclear elements (EhLINEs and EhSINEs respectively), are present on all chromosomes, and together occupy about 11% of the genome (Sharma et al., 2001; Lorenzi et al., 2008; Kaur et al., 2021). Another class of repeated DNAs are the ribosomal RNA genes, located exclusively on extrachromosomal circular molecules (Bhattacharya et al., 1998) rather than on linear chromosomes (Bagchi et al., 1999). The rDNA circles account for ∼10% of the genome. Due to their abundance, and the availability of their complete nucleotide sequence, early studies on *E. histolytica* DNA replication focused on the rDNA episome (Sehgal et al., 1994).

## 2 Replication of the ribosomal DNA episome of *E. histolytica*

In *E. histolytica* strain HM-1:IMSS the rRNA genes are located in the nucleus on extrachromosomal circles of size 24.5 kb. Each circle, present in about 200 copies, contains two rDNA transcription units of 5.8 kb each arranged as inverted repeats. They are separated by upstream and downstream intergenic spacers (IGS) (Bhattacharya et al., 1998). The rDNA circles analysed in different *E. histolytica* strains are very similar to the strain HM-1:IMSS. However, some circles contain one rDNA unit in place of two. These circular DNAs afford a convenient system for DNA replication studies.

Amongst unicellular eukaryotes the best studied nuclear plasmids are the *Saccharomyces cerevisiae* 2 μm plasmid and *Dictyostelium discoideum* plasmids. Much like bacterial plasmids where DNA replication initiates at a relatively fixed site, called the origin of replication (*ori*) (Couturier et al., 1988) here too, DNA replication initiates at fixed sites in the replicons. The yeast 2 μm plasmid initiates DNA replication at a unique site called the autonomously replicating sequence (ARS) which contains an 11-bp core and an AT-rich region to the 3′- side of the core (Volkert et al., 1989). The *D. discoideum* plasmids also initiate replication at a specific site that contains a 49-bp element present in three copies (Hughes et al., 1988).

The replication origin of the *E. histolytica* rDNA circles was mapped by using neutral/neutral two-dimensional gel electrophoresis**.** In this method **(**Brewer and Fangman, 1987) the replicon to be studied is digested with restriction enzymes. Electrophoretic migration of the restriction fragments is determined by gel electrophoresis in the first dimension based on size, and in the second dimension based on conformation. A restriction fragment that contains the *ori* where a replication fork starts and proceeds bidirectionally would have the shape of a bubble, while a fragment in which replication forks originating from the outside enter the fragment would contain Y arcs in place of bubbles. A fragment in which forks moving in opposite directions meet and terminate would have an X shape. These molecules can be readily distinguished by two-dimensional gel electrophoresis as their migration is retarded by varying degrees in the second dimension (Brewer and Fangman, 1987). The analysis of *E. histolytica* rDNA circles with this method showed that all fragments contained both simple Ys and bubbles, indicating the presence of multiple *oris* dispersed throughout the molecule, rather than a single fixed *ori* (Dhar et al., 1996)*.* This unexpected observation was further confirmed by electron microscopy. The rDNA circle was linearized with restriction enzymes and the location of replication bubbles from the cut ends was measured (Dhar et al., 1996). These data corroborated the gel electrophoresis study and showed that replication bubbles were located throughout the molecule and appeared with greater frequency within the rDNA transcription units.

Although multiple *oris* are unusual for plasmid replication, they are the norm for eukaryotic chromosomes which contain many potential *oris*. These are activated to initiate replication in a time and context-dependent manner (Bogan et al., 2000; Bell and Dutta, 2002; Kumar and Remus, 2016). Further analysis of the *E. histolytica* rDNA *oris* showed that here too, all potential origins are not used equally at all times (Ghosh et al., 2003). In exponentially growing cells the most commonly used *ori* mapped upstream of the rDNA transcription unit, close to the promoter of rRNA genes (Panigrahi et al., 2009), and terminated in the downstream intergenic spacer. However, when cells were starved of serum for 24 h, after which serum was replenished to resume replication in a synchronous manner, the early *oris* mapped within the rDNA transcription unit. At later times the *ori* in the promoter-proximal upstream IGS was activated, while the early *oris* in the transcription unit were silenced. These data suggest that while the upstream IGS is the preferred *ori* in exponentially growing cells, other potential *oris* exist. The latter are probably recruited when the upstream *ori* is not in an active state (e.g., in cells recovering from stress), but become silenced when the upstream *ori* is activated. Another significant observation was that the upstream IGS fragment of the rDNA episome did not show *ori* activity when it was located ectopically in an artificial plasmid. Other fragments of the rDNA episome could, however, function as *oris* in the ectopic location (Ghosh et al., 2003).

## 3 DNA replication enzymes

The availability of *E. histolytica* genome sequence in 2005 (Loftus et al., 2005), gave a major boost to biochemical studies on enzymes involved in major metabolic pathways (Table 1). *Entamoeba histolytica* genome sequence was searched for homologs of the canonical DNA polymerase enzymes. *Entamoeba histolytica* contains homologs of the replicative DNA polymerases α, δ, *ε* of the polymerase family B; lesion repair polymerases Rev1 and Rev3; and another translesion repair polymerase belonging to family A. In addition, there are five families of polymerases related to the family B2 encoded by DNA transposons and bacteriophage φ29 (Loftus et al., 2005; Lorenzi et al., 2010; Pastor-Palacios et al., 2010).

**TABLE 1:** Biochemically characterized replication and recombination proteins in *E. histolytica*.

Protein	Function	Unique Features	Comments	References
EhDNApolA	Translesion synthesis	Single family A DNA polymerase	Homologous genes in *E. invadens, E. dispar*	Pastor-Palacios et al. (2010)
EhDNApolB2	Polymerase, exonuclease, abasic site bypass	21 amino acid longer TPR2 motif	Related to DNA pol of Polinton-Maverick transposon	Herrera-Aguirre et al. (2010), Pastor-Palacios et al. (2010)
EhDNALigI	High fidelity DNA ligase; also involved in DNA repair	Shorter than human enzyme by 150 amino acids	Sole DNA ligase	Azauara-Liceaga et al. (2018)
EhPCNA	Interacts with EhDNALigI through PIP box	—	Connector loop and CTD required to stimulate EhDNALigI	Cordona-Felix et al. (2011), Trasvina-Arenas et al. (2017)
Helicase Cdc45 component	Part of replicative helicase	Has a shorter flexible insertion in DHH domain than human Cdc45	Full-length EhCdc45 crystallized due to shorter flexible insertion	Kurniawan et al. (2018)
EhRad51	Perform strand transfer and D-loop formation	Human Rad51 inhibitor B02 had no effect, DIDS inhibited activity	Could perform strand exchange over long DNA stretches	Lopez-Casamichana et al. (2008), Kelso et al. (2016)
EhDmc1	Perform strand transfer and D-loop formation	Known to express only in meiosis, but meiosis not demonstrated in *E. histolytica*	Could perform strand exchange over long DNA stretches	Kelso et al. (2015)

### 3.1 Family A DNA polymerase in *E. histolytica*

Family A DNA polymerases in eukaryotes are generally of three types. DNA polymerase γ of this family is the mitochondrial replicative DNA polymerase, while DNA Pol ν (Marini et al., 2003) and DNA Pol θ (Takata et al., 2006) are nuclear localized and are involved in translesion synthesis. A single putative DNA polymerase A member has been identified in *E. histolytica* by sequence homology with Klenow fragment. This EhDNApolA has been biochemically characterized and shown to be nuclear-localized and engaged in translesion synthesis (Pastor-Palacios et al., 2010). Homologous genes have also been identified in *Entamoeba invadens* (the reptilian parasite that is used as a model system for encystation studies) and *Entamoeba dispar* (the non-pathogenic species found in humans). A shared feature of the *Entamoeba* DNApolA members is the absence of a 3′-5′ exonuclease active site, while the polymerization site is well-conserved. As seen by homology modelling, EhDNApolA adopts a structure similar to the Klenow fragment bound to DNA, with the fingers, palm and thumb subdomains forming a DNA-binding cleft. EhDNApolA could incorporate dNTPs into an annealed primer-template, confirming its polymerase activity with kinetic constants similar to those of known family A DNA polymerases (Pastor-Palacios et al., 2010). It also had moderate strand displacement activity, but lacked 3′-5′ exonuclease activity. EhDNApolA had efficient translesion synthesis activity against a non-blocking lesion like that due to 8-oxoguanosine, and was also active against a blocking lesion like thymine glycol.

### 3.2 Family B2 DNA polymerase in *E. histolytica*

The archetypical Family B2 DNA polymerase is the bacteriophage φ29 enzyme (Rodriguez et al., 2005). In addition to the polymerization and 3′-5′ exonuclease domains, family B2 DNA polymerases contain two extra domains called terminal protein regions (TPR) 1 and 2 which confer processivity and strand displacement activities. The *E. histolytica* genome encodes four family B2 DNA polymerases (Herrera-Aguirre et al., 2010; Pastor-Palacios et al., 2010; Vicente et al., 2012). The closest orthologs of the *E. histolytica* enzymes are the DNA polymerases encoded by the DNA transposon class called Polinton-Maverick found in *E. invadens* (Kapitonov and Jurka, 2006; Pastor-Palacios et al., 2012). The amino acid sequences required for polymerization and exonuclease activities are well conserved in all 4 *E. histolytica* sequences (Pastor-Palacios et al., 2012). One of the 4 copies showing best match with φ29 enzyme was cloned and expressed. Amino acid sequence alignment of this copy (EhDNApolB2) with the φ29 enzyme showed 38% identity in the polymerase and exonuclease domains. The TPR2 motif was 21 amino acids longer in *E. histolytica*. In a structural model these 21 amino acids were shown to lie adjacent to the finger sub domain. The recombinant protein was shown to have polymerase and exonuclease activities. Enzyme activity was inhibited by aphidicolin, an inhibitor specific to family B DNA pols. EhDNApolB2 could also efficiently bypass lesions due to abasic sites or 8-oxoguanosine, although it could not bypass the strong block due to thymine glycol, which is expected from family B DNA polymerases. EhDNApolB2 achieves lesion bypass by incorporating dATP opposite the lesion. From deletion studies it was shown that the TPR2 motif was involved in efficient bypass of abasic sites, in addition to its known role in processivity and strand displacement.

### 3.3 The DNA ligase of *E. histolytica*

In contrast to vertebrates, *E. histolytica* encodes a single DNA ligase (EhDNAligI) which is similar to eukaryotic DNA ligase I (Azuara-Liceaga et al., 2018). It is an ATP-dependent DNA ligase of 685 amino acids with 35% identity to human DNA ligase I (Cardona-Felix et al., 2010). It is shorter than the human enzyme by 150 amino acids at the N-terminal. Being the sole DNA ligase, it is likely involved in both DNA replication in the maturation of Okazaki fragments, and in DNA repair processes. Biochemical analysis of recombinant EhDNAligI showed that it could carry out the essential steps in DNA ligation, namely, adenylation, binding to 5′-phosphorylated nicked DNA and sealing the nick. As expected of a family I DNA ligase it could ligate a RNA strand upstream of the nick, but not downstream of it. It was a high-fidelity DNA ligase and could discriminate 11 of the 12 possible mismatches on either side of the nick on the two DNA strands. The only mismatch efficiently ligated was the T:G mismatch at the 5′-phosphate strand (Azauara-Liceaga et al., 2018). This wobble base-pair mismatch is seen in ligases of *Escherichia coli* and *Thermus thermophilus* as well (Chauleau and Shuman, 2016). The possible involvement of EhDNAligI in DNA repair was suggested by various observations. In cells subjected to oxidative DNA damage EhDNAligI was shown to translocate to the nuclear periphery and co-localize with PCNA and the 8-oxoguanosine adduct. Following recovery from stress and elimination of 8-oxoguanosine, EhDNAligI was no longer concentrated at the nuclear periphery and was seen diffusely in the cytoplasm.

### 3.4 EhPCNA

PCNA is a highly conserved, essential accessory protein in DNA replication and repair. It serves as a DNA clamp; forms a homo trimeric ring around the DNA and slides along the DNA length. PCNA recruits a variety of proteins involved in DNA replication, repair and chromatin structure (González-Magaña and Blanco, 2020). It interacts with its protein partners via specific motifs, one of which is the PCNA-interacting peptide (PIP) box. The *E. histolytica* homolog of PCNA was identified by sequence search. Biochemical and structural studies with the recombinant EhPCNA showed that it formed a homotrimer and had a PIP box through which it interacted with EhDNAligI (Cordona-Felix et al., 2011). The interaction of EhDNAligI and EhPCNA was studied in more detail (Trasvina-Arenas et al., 2017). The N-terminal region of EhDNAligI is only 39 amino acids (compared with 262 amino acids in the human enzyme HsDNAligI), and is thought to be involved in protein-protein interactions. The PIP box is located at residues 2 to 9. Two deletion mutants of EhDNAligI were generated-one lacking the first 39 amino acids (∆PIP) and another lacking the first 58 amino acids in which, along with PIP, two putative alpha helices of the DNA binding domain (∆DBD) were deleted. Both deletion mutants could perform the first step of ligation reaction, that is self-adenylation, although the ∆DBD had reduced activity compared with full-length. ∆PIP could carry out the second step of ligation reaction, that is binding of the ligase to 5′- phosphate of the DNA nick with kinetics comparable to the full-length, and the increase in binding affinity in the presence of EhPCNA was also comparable; showing that PIP box of EhDNAligI was not needed for interaction with EhPCNA. Although ∆DBD did not bind to nicked DNA substrate, addition of EhPCNA could restore this capability. These data suggest that the initial interaction between EhDNAligI and EhPCNA occurs through the PIP box, and a second interaction takes place between the two proteins through a specific interface. Using affinity chromatography it was shown that EhPCNA could bind with both deletion mutants, showing that the two proteins interacted independent of the PIP box. Further it was shown that the nick-sealing activity of ∆DBD was restored by EhPCNA. The interdomain connector loop and C-terminal domain of EhPCNA was required for stimulating the activity of EhDNAligI. The C-terminal of EhPCNA probably allows allosteric transition of EhDNAligI from a spread-shape to a ring structure.

### 3.5 The Cdc45 component of *E. histolytica* replicative helicase

Successful DNA replication requires unwinding of the DNA duplex which is achieved by specialized replicative helicases. These are molecular motors that unwind DNA at the front of the replication fork, and translocate along single-stranded DNA using energy from nucleotide hydrolysis. In eukaryotes, replisome assembly at the ori starts with loading of the hexameric minichromosome maintenance complex (Mcm) 2–7. This is activated through a series of events, leading to the recruitment of cell division cycle 45 (Cdc45) and Go-Ichi-Ni-San (GINS) proteins, which together form the active replicative helicase Cdc45/Mcm2-7/GINS (CMG) holoenzyme. In model organisms, the molecular mechanism by which the replicative helicase operates is being revealed through single molecule studies (Spinks et al., 2021) and cryo electron microscopy (EM) (Kurniawan et al., 2018). It has been shown that Cdc45 and GINS bind to the outer surface of the N-tier ring of the hexameric Mcm2-7 complex to stabilize its closed conformation (Petojevic et al., 2015). This allows the ATPase motor of Mcm2-7 to catalyze translocation of CMG complex along the DNA strand. Orthologs of the replicative helicase proteins exist in *E. histolytica* but their biochemical properties have not been studied. However, the *E. histolytica* Cdc45 protein has been studied by cryo EM (Kurniawan et al., 2018). This study was motivated by the fact that it was difficult to crytallize full-length human Cdc45 due to the presence of a flexible insertion within the N-terminal DHH domain of this protein. Crystal structure could be obtained only after deleting an11-amino acid stretch from this region. The 543-residue, *E. histolytica* Cdc45 protein has a shorter flexible insertion in this region, making it possible to crystallize the intact protein. The *E. histolytica* Cdc45 consists of the conserved N-terminal DHH and C-terminal DHHA1 domains connected through an α-helical connector segment. Its structure could be solved to 1.66 Å resolution. There was overall similarity to human Cdc45, but the E. histolytica Cdc45 had several unique features. There was a distinct orientation of the C-terminal DHHA1 domain due to which there was disordering of the adjacent protruding α-helical segment implicated in DNA polymerase ɛ interactions. The authors suggest that Cdc45 could adopt multiple conformations and thereby regulate protein-protein interactions important in DNA replication.

## 4 DNA recombination in *E. histolytica*

Parasitic protists are continuously exposed to environmental toxins and host immune pressure, which can affect the stability of their genome. Homologous recombination (HR) is a conserved mechanism that can repair DNA damage due to double strand breaks (DSBs). These could occur due to genotoxic agents or during normal processes like meiotic division, or during DNA synthesis to enable bypass of lesions that block the replicative polymerase (Brandsma and Gent, 2012). HR is important to generate genetic diversity which parasites use to evade the host immune response (Deitsch et al., 1999; Conway et al., 2002). DSBs, caused by DNA damage or by the Spo11 endonuclease during meiosis, trigger HR (Keeney et al., 1997; San Filippo et al., 2008). DSBs are processed by the Mrn/Mrx complex, which includes Mre11p, to generate single strand tails with 3′-OH ends. The ssDNA forms a nucleoprotein filament in association with Rad51p and Dmc1p (Masson and West, 2001) which is stimulated by the Hop2-Mnd1 proteins to invade a homologous sequence and form a D-loop intermediate (Tsubouchi and Roeder, 2002). The Msh2 protein (which interacts with Mlh1p) is part of a complex involved in strand invasion. Subsequent steps lead to the capture of the second DSB end and the formation and resolution of Holliday junctions, promoted by the Msh4, Msh5 and Mer3 proteins (Hunter and Kleckner, 2001).

A search of the *E. histolytica* genome showed the presence of many HR-related genes. Some meiotic genes like Spo11, Dmc1, Mnd1, and many HR specific genes like Mlh1, Msh2, Rad21, and Rad51 are present in both *E. histolytica* and *E. invadens* (Bhattacharya et al., 2000; Ramesh et al., 2005; Malik et al., 2008; Singh et al., 2013; Kelso et al., 2016). HR-related genes were upregulated when DSBs were induced by UV-C irradiation (López-Casamichana et al., 2008) or when cells were subjected to growth stress (Singh et al., 2013). Further, HR could be directly demonstrated, and its efficiency increased during growth stress in *E. histolytica* and during encystation in *E. invadens* (Singh et al., 2013). These data suggest that HR operates in *Entamoeba* and could have critical roles in trophozoite growth and stage conversion.

### 4.1 Direct demonstration of HR in *E. histolytica*

The presence of a large number of HR-related genes in *E. histolytica* suggests that this conserved process should occur in this organism. However, the absence of markers for genetic selection, lack of any obvious sexual stages, and the inability to knock out genes by homologous recombination made it difficult to directly demonstrate HR in *E. histolytica* cells. Using an alternative approach based on recombination between inverted repeats it was possible to directly demonstrate the process of HR (Singh et al., 2013). *Entamoeba histolytica* and *E. invadens* cells were stably transfected with a plasmid containing two identical inverted repeats of 730 bp each, separated by a 1.6 kb fragment of luciferase (LUC) sequence. Recombination between the inverted repeats would cause flipping of the LUC sequence. This was measured by PCR using one primer from the LUC sequence and another from flanking sequence in the plasmid backbone. In the parental construct the two primers would be in the same orientation and would fail to give an amplicon. If recombination occurred between the inverted repeats the two primers would now be facing each other due to flipping of sequence, and a PCR product of expected size would be obtained. As an internal control a primer pair was designed from the LUC sequence which would give an amplicon in both the parental plasmid and that obtained post-transfection. This strategy showed that HR indeed takes place in *E. histolytica* cells, as an amplicon was obtained from the plasmid DNA of transfected cells but not from the original plasmid. The amplicons were sequenced to further demonstrate flipping of the sequence exactly at the expected recombination junction. Apart from cells grown under normal growth conditions HR was also checked in cells under different stress conditions, namely, serum starvation, heat stress, oxygen stress and UV-C irradiation. HR was activated in all stresses, by 1.4- to 2.7-fold. The same experimental protocol was used to check HR during cyst formation in *E. invadens* as well (Singh et al., 2013). Within 8 h of transfer of trophozoites to encystation medium recombination efficiency went up and remained high (3–4 fold) throughout encystation. Active recombination was also observed during excystation when the cysts obtained were transferred to fresh growth medium.

HR events were also demonstrated at chromosomal loci using the short interspersed nuclear element (SINE) sequences which are abundant in the *E. histolytica* genome. From the genome sequence database two SINE pairs were selected which were organized as inverted repeats in relatively close proximity on the same scaffold (Huntley et al., 2010). As in the plasmid study, recombined molecules could be detected here as well by using PCR primers that would give an amplicon only when recombination occurred between the inverted SINE repeats. HR occurred in exponentially growing cells and was enhanced under conditions of growth stress, concomitant with the upregulation of HR genes. Thus, inverted repeat sequences, either located on a plasmid or chromosomally, are capable of recombining in *E. histolytica*.

### 4.2 Transcriptional changes in HR genes during genotoxic stress in *E. histolytica*

Cells undergoing stress can suffer DNA damage, which is a trigger for HR. Several studies have looked at transcriptional changes in HR-related genes of *E. histolytica* in a variety of stresses.

#### 4.2.1 UV-irradiation-induced stress

HR was induced in *E. histolytica* trophozoites irradiated with 254 nm UV-C (López-Casamichana et al., 2008; Charcas-Lopez et al., 2014). The appearance of DSBs was shown by TUNEL and comet assays, and was also suggested by histone phosphorylation. It is known that DSBs induce phosphorylation of human histone H2AX at the serine residue located in the SQ motif at the C-terminus (Chen et al., 2000). The same SQ motif is present in *E. histolytica* histone H2A as well, since the canonical histone H2A sequence of *E. histolytica* has been replaced with H2AX (Sullivan et al., 2006). Two gene sequences homologous to H2AX have been identified in *E. histolytica* and both have the conserved SQ motif (Lopez-Casamichana et al., 2008). The amount of phosphorylated H2AX, measured by Western blot analysis increased 5-fold in *E. histolytica* cells after UV-C treatment for 10 min, indicating the presence of DNA DSBs. This system was used to look at transcriptional changes (measured by semi-quantitative RT-PCR) in *E. histolytica* cells responding to DNA DSBs induced by UV-C treatment. It was found that EhMre11, EhRad51, EhRad51c and EhRad52 genes were transcribed at a very low level in non-irradiated trophozoites, and their expression levels went up after UV-C treatment. EhRad51 levels went up 16-fold, 30 min after UV-C treatment. On the other hand, the expression levels of EhNbs1, EhRad54 and EhRad59 genes which were abundantly transcribed in untreated trophozoites, dropped after UV-C treatment (Lopez-Casamachina et al., 2008). Possible post-transcriptional regulation of these genes in UV-treated cells was not studied. This system was further studied to look at global transcriptional changes in UV-C treated cells, using cDNA microarrays (Weber et al., 2009). Most genes showed less than 2-fold change in transcription levels. Interestingly, the genes coding for Fe-S clusters-containing proteins were over-expressed, showing that these could be involved in adaptation to DNA damage. Several genes coding for cytoskeleton proteins were downregulated, suggesting that actin dynamics could be impaired after UV irradiation.

#### 4.2.2 Serum starvation-induced growth stress

Serum starvation induces a similar set of proteins as found during UV irradiation in HeLa cells (Glazer et al., 1989). Starvation is also known to trigger trophozoite to cyst conversion in Entamoeba, a process which could depend on meiotic recombination (Sanchez et al., 1994). To check for changes in expression of HR genes during serum starvation, exponentially growing *E. histolytica* cells were transferred to serum-deprived medium and starved for 24 h. Samples were removed at various time points and RNA levels of candidate genes were measured by qRT-PCR and northern hybridization (Singh et al., 2013). The analysed genes represented major steps of the recombination pathway, e.g., formation of DSBs (Spo11), sister chromatid pairing (Rad21), resection of ends following DSBs (Mre11), formation of nucleoprotein filament (Rad51, Dmc1), recombinase enhancement by stabilizing the presynaptic filament (Mnd1), mismatch repair during strand invasion (Msh2), and resolution of Holliday junctions (Mlh1). The expression levels of all genes went up in starved cells, and by 18 h of starvation the increase was between 1.2- to 5.5- fold. The relative expression of these genes was compared by Northern blot analysis. EhSpo11, EhMnd1, EhRad21 and EhMre11 were expressed at low levels in normal proliferating cells whereas EhDmc1 was expressed at moderate levels. These data showed that not only are homologues of HR genes present in *E. histolytica*, they are transcribed into mRNAs of the predicted size, and are upregulated during growth stress.

### 4.3 Transcriptional changes in HR and meiosis-specific genes during trophozoite to cyst conversion in *Entamoeba invadens*

*Entamoeba invadens* has been the model organism used to study encystation in *Entamoeba*, as it was not possible to obtain *E. histolytica* cysts in lab cultures. Exponentially growing *E. histolytica* trophozoites were transferred to encystation medium in which it typically took 72 h to complete the process of encystation. To monitor transcriptional changes in HR genes, cell samples were removed at various time points, and the RNA was analysed by qRT-PCR and Northern blotting (Singh et al., 2013). The HR genes studied were EiRad51, EiRad21, EiMlh1, EiMre11, and EiMsh2; whereas the meiosis-specific genes studied were EiSpo11A, EiSpo11B, EiDmc1 and EiMnd1. All the tested genes (except EiRad51) were upregulated during encystation. Expression levels went up 2- to 7.8-fold after 16–24 h of induction after which they began to decline. By 72 h, when encystation was complete, the expression levels of all genes had dropped. Northern analysis showed that *EiSPO11*a and *EiSPO11*b were poorly expressed in *E. invadens* trophozoites whereas *EiDMC1* was expressed at moderate levels.

### 4.4 Biochemical characterization of the HR-related proteins EhRad51 and EhDmc1

#### 4.4.1 EhRad51

Rad51 and Dmc1 are the eukaryotic orthologs of *E. coli* RecA recombinase that is responsible for homology search and strand exchange (Sung and Robberson, 1995). It forms the nucleoprotein filament which invades the homologous duplex and results in a displacement loop (D-loop). DNA synthesis is primed from the invading strand to restore damaged DNA. Rad51 is ubiquitously expressed whereas Dmc1 is specific to meiotic cells (Bishop et al., 1992). Sequences homologous to both Rad51 and Dmc1 are present in the *E. histolytica* genome. EhRad51 has been studied in detail. It encodes a protein of predicted molecular weight 40.3 kDa. The recombinant protein expressed in *E. coli* was biochemically characterized (Lopez-Casamichana et al., 2008). It could bind both ss- and ds-DNA. When incubated with a circular dsDNA and homologous ssDNA it could perform strand transfer and D-loop formation, thus demonstrating the canonical properties of Rad51 recombinase. Another study further characterized EhRad51 using a highly purified recombinant protein expressed in *E. coli* (Kelso et al., 2016). As seen with Rad51 from other organisms (Chi et al., 2006), EhRad51 could hydrolyse ATP, and the activity was stimulated in the presence of DNA, especially ssDNA. In keeping with this observation, EhRad51 was shown to bind DNA, with a strong preference for ssDNA. A nuclease protection assay was used to demonstrate the ability of EhRad51 to form a presynaptic filament in the presence of ATP. The non-hydrolyzable analogs of ATP, and surprisingly, even ADP could support filament formation, showing that nucleotide binding is required for this reaction but hydrolysis is not. Further, the ability of EhRad51 to perform strand exchange was demonstrated by using φX174 circular ssDNA along with φX174 linear dsDNA which is 5.4 kb. Strand exchange reaction was measured by the appearance of ds nicked circular DNA. This is formed by extension of the initial joint molecule by EhRad51 through its strand displacement activity (Kelso et al., 2016). Thus, EhRad51 could perform *in vitro* strand exchange over at least 5.4 kb EhRad51 could weakly catalyze the formation of D-loops, and this activity was stimulated 4-fold in the presence of calcium, as previously observed for human Rad51 (Bugreev and Mazin, 2004). D-loop formation was greatly enhanced by the addition of murine Hop2-Mnd1 which is a known accessory factor for Rad51 (Petukhova et al., 2005). This observation also shows the strong evolutionary conservation of Hop2-Mnd1 function.

#### 4.4.2 EhDmc1

Dmc1 has the same properties as Rad51, with the exception that it is expressed only in meiotic cells. A sequence homologous to Dmc1 has been identified by sequence search. The recombinant EhDmc1 protein has been purified and biochemically characterized (Kelso et al., 2015). Much like EhRad51, EhDmc1 was also found to catalyze strand exchange over long DNA stretches. This activity was stimulated by calcium and the accessory factor Hop2-Mnd1. Although meiosis has not been demonstrated in *E. histolytica*, EhDmc1 was found to express in proliferating cells of *E. histolytica*. This raises the question whether meiosis, indeed, occurs in *E. histolytica* trophozoites, or whether EhDmc1 has another unidentified role, for instance in maintaining the polyploid state of *E. histolytica* (Willhoeft and Tannich, 1999).

### 4.5 HR as a therapeutic drug target

DNA repair and recombination are essential for parasite growth, and for it to cope with environmental stresses and evade the host immune response. Thus, this process could be a potential target for new therapies.

Two of the known inhibitors of human Rad51, DIDS (4,4′-diisothiocyanostilbene-2,2′-disulfonic acid) and B02 were tested with EhRad51 (Kelso et al., 2016). While DIDS inhibited the strand exchange activity of EhRad51, B02 did not have any significant effect. The same was also seen with EhDmc1. DIDS inhibited EhDmc1, and, importantly, it could strongly inhibit encystation in *E. invadens* whereas it had no effect on cell proliferation. Although it is possible that DIDS could inhibit encystation through an independent mechanism, these data do support the view that HR may be essential during encystation in *Entamoeba*. In addition, independent of the mechanism of action, DIDS could be one of a promising class of molecules of therapeutic potential to reduce infectivity by inhibiting cyst formation. Although DIDS had an inhibitory effect on EhRad51 and EhDmc1, it cannot itself be used directly as a drug due to its high toxicity in humans (Ishida et al., 2009).

## 5 Discussion

DNA replication studies in *E. histolytica* started with the rDNA episome which, due to its small size and high copy number, was an attractive system. Unlike most other prokaryotic and eukaryotic plasmids, this episome did not initiate replication from a fixed origin. Rather it shared some hallmarks of eukaryotic chromosomal DNA replication where there are many potential origins, of which some are fired at a given time and context (Bell and Dutta, 2002). The *E. histolytica* rDNA episome also contained many potential origins, which were not used equally at all times (Dhar et al., 1996). In exponentially growing cells the most commonly used *ori* mapped upstream of the rDNA transcription unit, close to the promoter of rRNA genes. However, when cells were starved of serum and replication was resumed in a synchronous manner by serum replenishment, the early *oris* mapped within the rDNA transcription unit, while at later times the *ori* in the promoter-proximal upstream IGS was activated (Ghosh et al., 2003). It is interesting that the upstream *ori* mapped very close to the rDNA promoter. A similar pattern is observed in other eukaryotes and it has been suggested that binding of transcription factors could influence the initiation of DNA replication. In *Xenopus* oocytes when cells divide rapidly without transcription during early embryogenesis, replication initiates from multiple *oris* in the rDNA locus. At mid-blastula stage when rDNA transcription is activated, the *ori* shifts to the IGS (Hyrien et al., 1995).

Context-dependent *ori* usage has also been reported in the β-globin locus. Here the deletion of an upstream region called the locus control region (LCR) represses transcription of the locus and also the *ori* (Aladjem et al., 1998). However, when the β-globin *ori* is placed in an ectopic location, it can function without the LCR. Hence, in addition to the *ori* sequence, factors like chromatin organisation which could change in different genomic contexts may influence initiation. These data suggest that the pattern of replication initiation and *ori* usage seen in the *E. histolytica* rDNA episome has strong similarities with eukaryotic chromosomes rather than plasmids. This is possibly because episomal rRNA genes in various protozoa (Ravel-Chapuis et al., 1985; Clark and Cross, 1987) probably originate from chromosomal loci, and have thus retained the same regulatory signals. Rather than being arranged in hundreds to thousands of tandemly-repeated copies on linear chromosomes as in most eukaryotes, these protozoa maintain their rRNA genes on multi-copy plasmids that were probably ‘pinched off’ from chromosomal locations.

Homologs of most of the DNA replication and recombination proteins could be found in *E. histolytica* by genome sequence analysis. Many of these genes were cloned and expressed, and the purified proteins were analysed biochemically. Some of them, like the family B2 DNA polymerases, show interesting evolutionary origins. The closest orthologs of the *E. histolytica* Family B2 DNA polymerase enzymes are those encoded by the DNA transposon class called Polinton-Maverick found in *E. invadens* (Kapitonov and Jurka, 2006; Pastor-Palacios et al., 2012). The archetypical Family B2 DNA polymerase is the bacteriophage φ29 enzyme (Rodriguez et al., 2005). Compared with this enzyme the TPR2 motif (found in family B2 DNA polymerases) was 21 amino acids longer in *E. histolytica.* From deletion studies it was shown that the TPR2 motif was involved in efficient bypass of abasic sites, in addition to its known role in processivity and strand displacement. Thus, the *E. histolytica* enzyme seems to have acquired additional specificities through the extra 21 amino acid motif. It was possibly derived from a DNA transposon which is still found in the *E. invadens* genome, although very few copies of it exist in extant *E. histolytica*.

The presence of most of the conserved recombination-related genes in *E. histolytica* indicates that the process operates in this parasite. However, the absence of markers for genetic selection, lack of any obvious sexual stages, and the inability to knock out genes by homologous recombination made it difficult to experimentally demonstrate HR in *E. histolytica* cells. Several lines of evidence now point to the existence of HR in *Entamoeba*. These include differential expression of HR-related genes in response to DSBs induced by UV-C irradiation (López-Casamichana et al., 2008) or when cells were subjected to growth stress (Singh et al., 2013). Importantly, HR could be directly demonstrated during growth stress in *E. histolytica* and during encystation in *E. invadens* (Singh et al., 2013) using inverted repeat sequences. Detailed analysis of *E. invadens* cells undergoing encystation showed the upregulation of HR and meiotic genes at 16–24 h after transfer to encystation medium. This indicates that HR and meiotic recombination is likely to be important in conversion of the uninucleate trophozoite to tetra nucleate cyst. Although there is no direct evidence of DNA exchange in *E. invadens*, studies with *Giardia intestinalis* indicate that DNA exchange occurs following nuclear fusion (karyogamy) during encystation in this organism (Poxleitner et al., 2008). It has been suggested that the presence of homologous gene sequences corresponding to the core meiotic machinery in *Giardia* and other protists (*Entamoeba*, *Plasmodium*, *Trypanosoma* and *Leishmania*) is indicative of an ancestral origin of meiosis and there is a central role of this process in all extant eukaryotes (Ramesh et al., 2005).

Although HR does operate in *E. histolytica*, its efficiency during normal growth may be low. That could be a possible reason for difficulty in obtaining gene knockouts in *E. histolytica*. The observation that HR is activated in *E. histolytica* cells immediately after exposure to a variety of stress conditions could be exploited to obtain gene knock-out in this organism (Singh et al., 2013). Efficiency of gene knock-out could be enhanced if the cells transfected with the suitable DNA construct were exposed to a brief duration of stress. HR is an essential process for parasite growth and differentiation, making it an attractive drug target. However, since HR is a highly conserved process, and is also essential for the host, the challenge would be to find species-specific inhibitors of HR proteins that selectively inhibit *E. histolytica* HR (Kelso et al., 2017). The fact that the human HR inhibitor B02 had no effect on *E. histolytica* HR shows that it should be possible to find *E. histolytica* -specific inhibitors as well (Kelso et al., 2016). High throughput screens could be set up using recombinant EhRad51 to look for small molecule inhibitors of the *E. histolytica* enzyme which do not inhibit the human enzyme. Future studies may provide structural information about *E. histolytica* recombinases, which could be used to design *Entamoeba*-specific inhibitor molecules.
